# Elevated allostatic load early in the course of schizophrenia

**DOI:** 10.1038/s41398-018-0299-z

**Published:** 2018-11-12

**Authors:** Anya Savransky, Joshua Chiappelli, Feven Fisseha, Krista M. Wisner, Du Xiaoming, S. Milad Mirmomen, Aaron D. Jones, Bhim M. Adhikari, Heather A. Bruce, Laura M. Rowland, L. Elliot Hong

**Affiliations:** Department of Psychiatry, Maryland Psychiatric Research Center, University of Maryland School of Medicine, Baltimore, MD USA

## Abstract

Stress plays a significant role in schizophrenia from disease onset to exacerbation of psychotic symptoms. Allostatic load (AL) is a measure of cumulative stress to the organism. This study is an extension of our previous work on AL and its relationship to brain structures. Here, we further determined whether elevated AL is a function of illness chronicity, or if it is already present early in the course of schizophrenia. AL was compared in schizophrenia patients early in the illness (within 5 years of disease onset), patients with chronic schizophrenia (more than 5 years of illness), and two groups of healthy controls that were age-and sex-matched to the two patient groups. This work is presented with an expanded sample and includes about two-thirds of the participants who were previously reported. We found that patients with early psychosis had significantly elevated AL score compared with their age-matched controls (*p* = 0.005). Chronic course patients also had elevated AL compared with age-matched controls (*p* = 0.003). Immune and stress hormone AL subcomponents were nominally higher in early-stage patients compared with controls (*p* = 0.005 and 0.04, respectively). Greater AL was also associated with more severe positive psychotic symptoms in early-stage patients (r = 0.54, *p* = 0.01). Elevated levels of allostatic load are already present in the early years of the schizophrenia illness, particularly in patients with more severe psychotic symptoms. AL may be a useful evaluation for the need of early intervention on psychosomatic comorbidity.

## Introduction

Stress plays an important role in psychosis. Stressful events often precede the onset of schizophrenia and episodes of psychosis symptom exacerbation^1–3^. Patients with first episode psychosis (FEP) showed dysregulation of stress responses under several environmental or laboratory conditions^4–7^. Similar findings were reported in individuals who are at high risk for psychosis or in first episode medication-free patients^8–10^, suggesting that hypothalamic–pituitary–adrenal (HPA) axis abnormalities start early in the course of schizophrenia, are not necessarily secondary to medication treatment, and are potentially part of the disease pathophysiology.

Patients with FEP and in early course of the illness also exhibit increased plasma levels of pro-inflammatory cytokines, such as interleukin 6, interleukin 1β, and C-reactive protein (CRP),^11,12^ elevated cardiovascular and metabolic abnormalities^13–17^, and maladaptive sympathetic nervous system responses^18^. However, there are discrepant findings regarding whether cardiovascular and metabolic disturbances occur in early stage of schizophrenia, as some studies found no evidence of significant increase^19–22^ or even lower cardio-metabolic risk^21,23^, whereas others report elevated rates of being overweight and impaired fasting glucose^24^. Antipsychotic medication use also substantially contributes to some of these abnormalities^21^. The question is whether the risks leading to abnormalities in stress, inflammatory, cardiovascular, and metabolic systems may have already started in the early years of the illness. This question has clinical urgency as patients with schizophrenia exhibit reduced life expectancy by 15–25 years compared with the general population, with cardiovascular and metabolic diseases are among the top causes of death^25,26^.

Characterizing the underlying mechanism of the diverse somatic dysfunctions in patients at such young age is challenging. One shared underlying factor for these dysfunctions may be the effects of cumulative stress. The body’s adaptive responses that promotes successful coping with repeated stressors can be described as allostasis^27^. Whereas, the concept of allostatic load (AL) refers to the “wear and tear” of the body after repeated maladaptive responses to adverse physiological and psychological stressors^28^. Previously, we found that patients with schizophrenia show elevated AL, and this is related to cortical and white matter abnormalities in the brain^29–31^. A recent study investigating AL in older-age “first episode” psychosis patients (averaged 33 years of age) also found elevated AL^32^. In the current study, we expanded on the original sample by including additional patients in the early course of the illness, as well as those in the chronic stage of illness, and re-examined whether AL is already significantly elevated within the first 5 years after the onset of symptoms. The rationale to focus on the first 5 years is that patients often experience severe functional decline in this early stage but then stabilize at a more chronic course in subsequent years^33^.

## Methods

### Participants

This study included 58 patients with schizophrenia spectrum disorders (SSD) (21 early and 37 chronic) and 34 healthy controls (14 younger and 20 older age- and sex-matched to each patient group). The patients (48 with schizophrenia and 10 with schizoaffective disorder diagnoses) were recruited from the Maryland Psychiatric Research Center and several neighboring mental health clinics. Healthy control participants were recruited through local media advertisements. Structured Clinical Interview for DSM-4 or -5 was used to confirm or exclude psychiatric diagnosis. Exclusion criteria included history of neurological conditions or head trauma with cognitive sequelae, or active and uncontrolled medical conditions. Participants with substance abuse and dependence other than nicotine were excluded. Healthy controls had no current Axis I psychiatric diagnosis. Psychiatric symptoms in the patients were assessed using the Brief Psychiatric Rating Scale (BPRS)^34^. To reduce cyclic hormonal effects in females, AL was measured within the first 10 days of their menstrual cycle. The AL data in about two-third of the participants were reported in previous studies on relationship to brain structures.^29–31^All participants gave written informed consent as approved by the University of Maryland IRB.

### Allostatic load assessment

The measure of AL was generated from 13 biomarkers: resting systolic blood pressure (SBP), diastolic blood pressure (DBP), and heart rate; body mass index (BMI) and waist-hip ratio; blood levels of high-density lipoprotein (HDL) cholesterol, total cholesterol, glycosylated hemoglobin (HbA1c), CRP, and dehydroepiandrosterone (DHEA); and 12-h overnight urine epinephrine, norepinephrine, and cortisol. A detailed description of the AL measure collection procedures can be found in our previous publications.^29-31^Adherent to previously reported methodology for calculation of AL index^35^, we identified the 25th and 75th percentile values of each of the 13 biomarkers for the respective control sample of each age period. Participants who had a biomarker value greater or equal to 75th percentile (or less than or equal to the 25th percentile for HDL and DHEA) received a score of 1 for that specific biomarker. Participants taking any hypoglycemic agents were automatically given a score of 1 for HbA1c; participants taking any antihypertensive medications were given a score of 1 for SBP; and participants taking any lipid-lowering medications were given a score of 1 for total cholesterol. The sum of biomarker values was computed such that the AL index score ranged from 0 to 13. To explore whether one or more of cardiovascular, metabolic, inflammation, and/or stress-related hormone subcomponents within the AL construct is more affected in the early course of schizophrenia, we divided the 13 AL measures into 4 subcomponents: cardiovascular (SBP, DBP, resting heart rate), metabolic (BMI, waist to hip ratio, HDL cholesterol, total cholesterol, hemoglobin HbA1c), inflammation (CRP), and stress-related hormones (epinephrine, norepinephrine, cortisol, DHEA), computed as the average of the scaled biomarkers.

The measures included in the AL index have considerable overlap with measures used to define metabolic syndrome. To determine whether the AL index was primarily driven by the presence of metabolic syndrome, the NCEP ATP III definition for metabolic syndrome^36^ was adapted to define metabolic syndrome: (1) HbA1c > 6.5 or taking hypoglycemic medication; (2) waist circumference > 40 inches for males or > 35 inches for females; (3) HDL < 40 for males, or < 50 for females; (4) SBP > 130, DBP > 85, or taking hypotensive medication. Any participant met more than two of the above criteria are considered having metabolic syndrome.

### Statistical analysis

None of the measures were significantly deviated from normal distribution as determined by Kolmogorov–Smirnov tests. Patient-control group differences in AL were compared using univariate ANOVAs with age and sex as covariates. Bonferroni corrections for multiple patient-control group comparisons were applied to AL subcomponent analyses, such that only group comparisons with *p* < 0.006 (0.05/4 subcomponents x 2 age cohorts) were considered significant. Pearson’s correlation was used to examine the relationship between AL and BPRS total and psychosis subscale scores.

## Results

### Allostatic load index

**Fig. 1:**
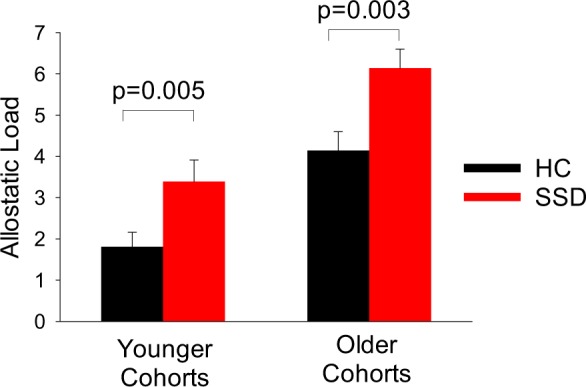
Allostatic load (AL) in schizophrenia spectrum disorder patients (SSD) and healthy controls (HC) in the younger and the older cohorts

Age (*p* = 0.97) and sex (*p* = 0.35) were frequency matched between patients who experienced 5 years or less of psychosis since disease onset (*n* = 21) and controls of the younger age cohort. These patients had elevated AL compared with controls (mean (SD): 3.39 (2.12) vs. 1.81 (1.33), F(1, 33) = 6.22, *p* = 0.018). Including age and sex as covariates, early-stage patients showed even numerically higher AL compared with controls (F = 9.17, *p* = 0.005) (Fig. 1).

Age (*p* = 0.94) and sex (*p* = 0.26) were also frequency matched between patients and controls of older age cohorts, where patients with the illness duration of more than 5 years (*n* = 37) also had higher AL compared with controls (6.14 (2.82) vs. 4.14 (2.31), F(1, 55) = 7.34, *p* = 0.009). After adjusting for age and sex, chronic patients also showed numerically even higher AL elevation compared with controls (F = 9.40, *p* = 0.003) (Fig. 1). The relationship between AL and smoking was also assessed. No diagnosis by smoking (all *p* > 0.31) or smoking by age interaction (all *p* > 0.79) were found in either groups. Examining the younger SSD and chronic groups revealed no diagnosis by smoking or smoking by age interaction in either groups (all *p* > 0.05).

As expected and similar to that previously reported^29–31^, the AL index score was significantly higher in patients compared with controls in the combined sample without (F(1, 90) = 11.52, *p* = 0.001) and with (F(1, 88) = 15.65, *p* < 0.001) the age and sex covariates.

### Relationship to sex

Stress-related findings are often sex-specific, so the data were analyzed in each sex separately. In the combined sample, AL was significantly different between patients and controls in male (5.02 (3.00) vs. 3.24 (2.60), respectively; t = −2.38, *p* = 0.02) and in female (5.46 (2.67) vs. 3.10 (1.76), t = −2.95, *p* = 0.006) groups. We also measured estradiol and progesterone levels, but these were not significantly different in the overall sample between patients and controls (Table 1).

**Table 1:** Demographics and clinical assessments in the combined patient and control samples

	**Schizophrenia (*n*** = 58)	**Healthy controls (*n*** = 34)	**F or χ** ^**2**^ **statistic**	***p*** **-value**
Age (years)	36.13 (14.33)	35.26 (14.03)	0.08	0.78
Sex (male/female)	41/17	20/14	1.35	0.26
Smoker/nonsmoker	19/39	7/27	1.57	0.24
BPRS total score	33.58 (9.63)	n/a	n/a	n/a
BPRS-positive symptoms	7.37 (4.09)	n/a	n/a	n/a
Age of onset (years)	19.85 ± 5.9	n/a	n/a	n/a
Metabolic syndrome (%)	21(36%)	4 (12%)	6.01	0.01
Estradiol levels	32.51 (35.61)	32.81 (40.12)	0.001	0.97
Progesterone levels	0.56 (0.36)	0.56 (0.32)	0.00	0.99
Allostatic load	5.15 (2.89)	3.18 (2.26)	15.65	< 0.001

Data were given in mean (standard deviation); *BPRS*brief psychiatric rating scale; statistics for AL include age and sex as covariates

### Subsystem markers

**Fig. 2:**
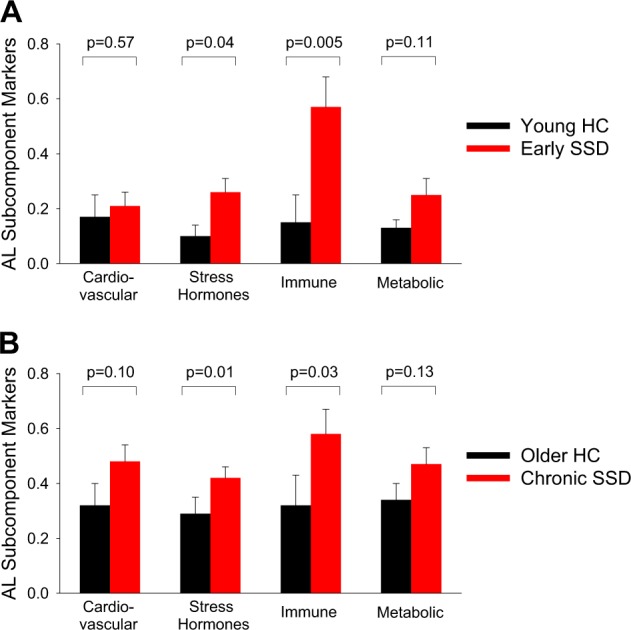
Cardiovascular, stress hormones, inflammatory, and metabolic subcomponents of allostatic load (AL) in schizophrenia spectrum disorder patients (SSD) and healthy controls (HC) in the younger (**a**) and the older (**b**) cohorts

We explored whether one or more of the cardiovascular, metabolic, inflammation, and/or stress-related hormone subcomponents may play a dominant role in the high AL in SSD (Fig. 2). The subcomponent calculations are from the categorical rating approach in AL calculation (see Methods section), and the divisions are approximations and some measures can be justified to be grouped differently. Out of all subcomponents, after controlling for age and sex, early-stage patients showed elevated stress hormone (F(1, 26) = 4.50 *p* = 0.04) and immune subcomponents (F(1, 30) = 9.25, *p* = 0.005), with the immune subcomponent to be significantly elevated after Bonferroni correction compared with the age-matched control group. Chronic patients also showed elevated stress hormone (F(1, 46) = 6.84, *p* = 0.01) and immune (F(1, 49) = 5.04, *p* = 0.03) subcomponents compared with their age-matched controls; none was significant after Bonferroni correction for multiple comparisons. Therefore, the only significant finding was significantly elevated immune subcomponent in the early-stage patients. The cardiovascular and the metabolic subcomponents were also higher in the patients but not significantly different between patients and controls in either age group. Actual measurement values were given in Table 2.

**Table 2:** Raw mean (SD) values of individual measures in the younger and older cohorts

	**Younger cohorts**		***p*** **-value**	**Older cohorts**		***p*** **-value**
	**Patients (*n*** = 21)	**Controls (*n*** = 14)		**Patients (*n*** = 37)	**Controls (*n*** = 20)	
Age	23.41 (4.34)	23.36 (3.61)	0.97	43.35 (12.91)	43.59 (12.46)	0.95
Sex (M:F)	16:5	9:5	0.47	25:12	11:9	0.39
*Cardiovascular*						
Resting SBP (mmHg)	109.12 (10.37)	111.36 (11.29)	0.21	114.86 (12.35)	118.18 (13.69)	0.25
Resting DBP (mmHg)	66.02 (7.92)	69.57 (6.72)	0.12	74.85 (8.26)	72.49 (10.40)	0.46
Resting heart rate (beats per minute)	72.79 (14.19)	69.43 (8.04)	0.30	81.55 (17.48)	66.46 (11.47)	0.002
*Metabolic – lipids*						
BMI (kg/m^2^)	27.05 (4.64)	26.56 (6.55)	0.57	30.22 (6.72)	28.31 (4.46)	0.23
Waist-hip ratio	0.88 (0.07)	0.85 (0.06)	0.13	0.98 (0.23)	0.90 (0.08)	0.14
HDL cholesterol (mg/dL)	52.05 (15.77)	52.43 (13.04)	0.94	52.57 (25.53)	57.28 (18.29)	0.51
Total cholesterol (mg/dL)	184.00 (38.87)	152.57 (38.04)	0.01	167.35 (33.49)	198.50 (39.43)	0.005
*Metabolic - glucose metabolism*						
Glycosylated hemoglobin (HbA1c)	5.37 (0.25)	5.39 (0.19)	0.99	6.04 (1.19)	5.58 (0.27)	0.08
*Inflammation*						
CRP (mg/L)	3.27 (4.68)	1.71 (2.59)	0.06	4.39 (4.69)	1.55 (1.65)	0.02
*Sympathetic nervous system*						
Urine epinephrine (ug/g creatine)	4.95 (3.38)	3.62 (2.82)	0.21	5.04 (3.59)	3.78 (2.45)	0.13
Urine norepinephreine (ug/g creatine)	26.13 (14.31)	21.33 (7.98)	0.32	46.48 (28.26)	25.88 (14.67)	0.004
*HPA axis*						
Urine cortisol (ug/g creatine)	10.47 (7.14)	11.27 (4.14)	0.67	12.91 (9.55)	21.64 (22.47)	0.10
Blood DHEA (ug/dL)	334.98(154.78)	289.78 (177.94)	0.62	219.16 (119.69)	208.89 (155.24)	0.87

*SBP* systolic blood pressure, *DBP* diastolic blood pressure, *BMI* body mass index, *HDL* high-density lipoprotein, *HbA1c*  hemoglobin A1c, *CRP* C- reactive protein, *HPA* hypothalamic–pituitary–adrenal, *DHEA*  dehydroepiandrosterone

Data in this table are raw values of each measure. Allostatic load was calculated as categorical data based on the 75% (25% for HDL cholesterol and dehydroepiandrosterone) cutoff for normality calculated by the values of the age-matched controls. Statistics given in the table was based on the categorical data

We also examined whether AL in both younger and older groups may be driven by the presence of metabolic syndrome. In the combined sample sample, 12% of subjects in the control group and 36% in the patient group met the criteria for metabolic syndrome. After covarying for metabolic syndrome, there was still a significant elevation of AL in the early-stage patients (F(1, 32) = 8.21, *p* = 0.007), but, interestingly, this was no longer significant in the chronic patients (F(1, 51) = 0.86, *p* = 0.36), each compared with the respective control groups.

### Clinical correlates of allostatic load

Greater AL was significantly associated with positive psychotic symptoms as measured by BPRS in early-stage patients (r = 0.54, *p* = 0.01), but not in chronic patients (r = 0.19, *p* = 0.29). Higher AL was correlated with earlier age of onset in younger patients at a trend level (r = −0.42, *p* = 0.06), but not in older patients (r = 0.02, *p* = 0.95). AL was not significantly associated with chlorpromazine (CPZ) equivalent dosage of antipsychotic medication in either group (all *p* > 0.05). We also assessed the relationship between AL and the duration of illness, which was not significant in either early SSD (*p* = 0.92) or chronic (*p* = 0.21) patients.

## Discussion

In this study, we found that patients with SSD within the first 5 years of psychosis onset already had significantly higher AL compared to their age-matched controls, which appeared not to be driven primarily by metabolic syndrome alone. Chronic patients also showed significantly elevated AL compared with age-matched controls, and the high AL in the more chronic patients appeared to be driven more by metabolic syndrome. These results support the hypothesis that AL elevation is already present in the early years of the illness.

A study investigating AL in older (mean age 32.96 years) FEP patients showed elevated AL^32^. This FEP sample was unique as the patients were not on antipsychotic medications, being about 10 years older than typical FEP cohorts, and AL decreased after 12 weeks of treatment^32^. As the patients were acutely ill during the initial assessment, it remains to be determined whether the psychosis reduction or antipsychotic medications per se were related to the AL changes. In the current study, patients had a stringent definition of being within the first 5 years of the course of psychosis, clinically stable, and the mean age of 23.41 was more representative of the typical age range of FEP patients. Overall, these data were consistent, and suggested that AL increases can be present in the early course of psychosis.

Schizophrenia spectrum disorder patients in the US have 28.5 years of shorter lifespan compared with the general population^25^. After accounting for suicide and other unnatural death, 85% of the deaths were natural causes from medical illnesses^25^. The risks of elevated cardiovascular and metabolic diseases in schizophrenia are well-known, and are further increased in aging patients with longer illness duration^19,37^. Understanding the complex sources of the high rate of medical conditions and the shorter lifespan in SSD is an urgent public health issue^38–40^. In this regard, we found that abnormal cardiovascular and metabolic subcomponents alone did not stand out as more prominent than other subcomponents in explaining elevated AL. This may be consistent with some literature, which showed that while mortality rate in early schizophrenia is already very high, metabolic and cardiovascular causes may not significantly contribute to the high mortality at this stage^41,42^. Covarying the metabolic syndrome also did not diminish the elevated AL in the early-stage patients, suggesting that elevated cumulative stress in the early course of illness may not be driven primarily by the presence of metabolic syndrome. Instead, cardiovascular and metabolic factors, when combined with stress hormone and immune factors yielded a robust AL abnormality even in the early course of schizophrenia. Therefore, most of the AL measures showed some levels of abnormalities in early stage of the illness but by each subcomponent the findings are not as robust as the overall AL index that aggregated these multisystem measures.

AL represents the effects from a cumulative burden of stress in individuals, and therefore the entire set of biomarkers may be more valid, where elevated AL is likely due to multiple mechanisms associated with maladaptive response to stress, resulting in multisystem imbalances even at the early years of schizophrenia. The significant correlation between AL and severity of positive symptoms in the early course patients (r = 0.54, *p* = 0.01) may further suggest that psychosis is either directly related to greater experience of stress, or indirectly associated with the underlying mechanisms of the maladaptive response to stress. This finding is also consistent with previous studies on first episode patients and individuals with clinical high risk where greater cortisol levels are associated with more severe positive symptoms^43^.

Indeed, some studies showed increased cortisol levels in individuals who are at risk for psychosis and in early psychosis and in non-medicated patients^8,44^, but not in other studies^45^. In our study, neither early nor chronic patients had significantly elevated levels of overnight urinary cortisol. Rather, the composite stress hormone subcomponent consisting of overnight cortisol, epinephrine, norepinephrine, and DHEA was nominally elevated in the early-stage patient group (*p* = 0.04), and this finding was replicable in the chronic patient group (*p* = 0.01). Findings suggest that elevated stress-related hormones at the overnight resting state are a consistent finding in early to chronic courses of schizophrenia. Similarly, we also observed elevated immune subcomponent (CRP) in early stage (*p* = 0.005) and chronic (*p* = 0.03) patients. CRP is an acute phase reactant used as a non-specific global measure for inflammation. Low-grade CRP elevation is a consistent finding in both first episode and chronic schizophrenia patients that cannot be attributed to antipsychotic treatment^12^.

On cardiovascular measures, several recent studies suggested that first episode patients show no elevation in 10-year cardiovascular diseases or metabolic syndrome rates compared with their age-matched peers^19,21^. Our data also did not support that parameters of cardiovascular and metabolic risk are particularly significantly elevated in early course of the illness.

Limitations of the study included that all but four of the patients were on antipsychotic medications at the time of the study. Antipsychotics can normalize diurnal cortisol hypersecretion^7,46^ and decrease cortisol levels,^47,48^ and even a single dose of second-generation antipsychotics can reduce serum cortisol level^49^. However, these antipsychotic medication effects should not explain the elevated stress-related hormone subcomponent, and high AL in patients at the early years of the illness except that they may have dampened the elevations of the stress subcomponent. More specific antipsychotic medication like aripiprizole, quetiapine, and olanzapine have been studied for their specific effects on decreasing or increasing norepinephrine releases,^50,51^ although to what extent these effects were related to the resting overnight urinary norepinephrine levels require additional studies. The modest sample size here did not permit analysis on specific effects from each antipsychotic medication. The effect sizes of AL in the early versus later disease course patients were similar, which did not support that cumulative dose of antipsychotics was the primary reason for the high AL. Still, the use of antipsychotic medications can have effects on cardiovascular and metabolic factors^52,53^. Another limitation is that our data were cross-sectional, and our analysis included the first five years of the onset of psychosis. A more narrow approach could be to examine AL in the initial month(s) of psychosis onset, ideally starting in patients with no or minimal antipsychotic medication exposures, followed by longitudinal follow-up of the cases. Such an approach may provide more conclusive evidence of whether high AL begins at disease onset versus develops within the first few years of the disease.

In conclusion, this study provided new evidence of a significantly elevated AL even in the first 5 years of the illness course of SSD. We found that subcomponents separately measuring immune, stress hormone, cardiovascular or metabolic factors were only weakly to modestly elevated in schizophrenia, but the overall AL metric that aggregates these components yielded significant disease effects even with a modest sample of early-stage young patients. AL may provide a theoretically driven, empirically applicable construct for supporting early risk identification, prevention, and treatment of somatic comorbidity in patients with SSD.
